# Vaccine Acceptance, Knowledge, Attitude and Practices Regarding the COVID-19 Pandemic: Cross-Sectional Study among Dentists in Trinidad and Tobago

**DOI:** 10.3390/dj11030086

**Published:** 2023-03-20

**Authors:** Reisha Rafeek, Bidyadhar Sa, William Smith

**Affiliations:** 1 School of Dentistry, Faculty of Medical Sciences, The University of the West Indies, St. Augustine, Trinidad and Tobago; 2 Centre for Medical Sciences Education, Faculty of Medical Sciences, The University of the West Indies, St. Augustine, Trinidad and Tobago

**Keywords:** COVID-19, vaccine acceptance, knowledge, attitude, practice, dentist

## Abstract

Background: This study’s aim was to assess Trinidad and Tobago dentists’ vaccine acceptance, knowledge, attitude and practices regarding the COVID-19 pandemic. Methods: All dentists registered with the Trinidad and Tobago Dental Association were invited to complete an online anonymous questionnaire between June and October 2021. Results: A total of 46.2% of dentists responded. The majority of respondents had excellent knowledge of COVID-19 (94.8%), use of personal protective equipment (98.7%) and N95 masks (93.5%), but had poor knowledge about the reuse of N95 masks (27.5%). A total of 34.9% were comfortable providing emergency care to positive or suspected cases of COVID-19, and 64.5% were afraid of becoming infected from a patient. PPE usage was reported at 97.4% and 67.3% for N95 masks. All surfaces of waiting areas were disinfected every 2 h by 59.2%. A total of 90.8% agreed to be vaccinated straight away if a vaccine were made available. Conclusion: Dentists in Trinidad and Tobago have good levels of knowledge, attitude, practices regarding COVID-19. Dentists also have high levels of vaccine acceptance and can play a role in advocating for the COVID-19 vaccine.

## 1. Introduction

The COVID-19 pandemic has had a profound impact globally, with 655,764,845 cases and 6,674,172 deaths reported to date [1]. Coronavirus is transmitted mainly during close contact from human to human through respiratory droplets. As such, the risk of cross-infection between dentists and patients is very high, due to potentially contaminated aerosol generated by high-speed rotary instruments and ultrasonic scalers used on COVID-19 positive patients [2,3].

In Trinidad and Tobago, a twin-island state in the Caribbean, the first case was reported on 12 March and on 15 March 2020, in which the government mandated a stay-at-home order for everyone except those working in essential services. The Ministry of Health (MOH) issued public guidelines based on CDC recommendations including quarantine, physical distancing, hand hygiene and mask wearing. The provision of emergency dental treatment was allowed under essential services in the Public Health Ordinance legal notice. However, no aerosol generation was allowed [4]. Currently, there have been 4277 deaths and 185,882 infections in Trinidad and Tobago [5].

There was a lot of uncertainty regarding the safe practice of dentistry under these conditions. The Dental Council of Trinidad and Tobago (DCTT) issued guidance for the reopening of dental practices in June 2020 that included the use of personal protective equipment (PPE), patient preparation and disinfection in the work environment. Later, The Centre for Disease Control (CDC) published recommendations for strict infection control procedures in dental settings [6].

There was a global race to produce COVID-19 vaccines, and the WHO provided Emergency Use Listing (EUL) to several vaccines such as Pfizer/BioNTech and COVISHIELD in December 2020, AstraZeneca in February 2021, Johnson and Johnson in March 2021, Moderna in April 2021, Sinopharm in May 2021 and Sinovac in June 2021 [7].

The Astra Zeneca vaccine was the first vaccine available in Trinidad and Tobago from March 2021 via the COVAX facility, though only in limited quantities [8]. It was offered free of charge to frontline healthcare workers (HCWs), the elderly over 65 years and those with underlying medical conditions as part of the government program first rollout of vaccinations. By July 2021, the Sinopharm vaccine was made available, and all HCWs and the general population were encouraged to become vaccinated at no cost [8]. Immunization remained voluntary and was not mandated for either HCWs or the general population.

At the time of the conceptualization of the study, few studies were published with regard to dentists’ knowledge, attitudes or practice, and COVID-19—none of which were from the Caribbean region. These publications originated from Jordan [9], Lebanon [10] and two multi-country surveys [11,12]. Vaccine hesitancy remained an issue amongst healthcare workers [13], and there was scarce information regarding the level of vaccine hesitancy amongst dentists. This gap in the literature made it difficult to guide the development of effective recommendations to control the spread of COVID-19 among the dental profession. This study aimed to assess Trinidad and Tobago dentists’ vaccine acceptance, knowledge, attitude and practices regarding the COVID-19 pandemic. The null hypothesis is that there is no difference in knowledge attitude or practice between dentists’ sex, specialty, experience or type of practice.

## 2. Materials and Methods

### 2.1. Study Design and Population

A cross-sectional study design was chosen utilizing an anonymous online questionnaire. The population chosen was all dentists registered with the Trinidad and Tobago Dental Association (TTDA). The questionnaire was developed by the authors based on the published literature [7,9,10,11]. The structured questionnaire consisted of 50 questions in 6 sections: Section A was reserved to demographic data. Section B consisted of questions related to dentists’ knowledge of COVID-19, and with answers for these questions composed of “correct”, “incorrect” and “not sure”. Section C related to dentists’ attitudes towards treatment of patients with COVID-19 and other concerns, with the answers to these questions composed of “yes” or “no”. Section D comprised of questions of dentists’ practices, with the answers to these questions composed of “always”, “sometimes” or “not at all”. Section E related to the source of information for dentists on COVID-19, and Section F related to dentists’ attitudes towards vaccines, with one question requiring a “yes” or “no” response and the other questions required answers of “agree”, “disagree” or “undecided”. It was pilot-tested on five dentists to check the clarity of all items.

Sample size calculation was performed using the online Raosoft sample size calculator designed specifically for population surveys (Raosoft, Inc.,Seattle, USA). The sample size was calculated at 179 with a confidence level of 95% and a 5% margin of error for the 331 dentists registered with the TTDA.

Ethical approval of the study was obtained from the Campus Research Ethics Committee of the University of the West Indies, St. Augustine campus (CREC-SA.1007/05/2021).

An invitation for voluntary participation in the study was sent via email and WhatsApp to all 331 dentists of the TTDA. The invitation included a link to the study portal which gave a brief background and objectives of the study, assurances of anonymity and confidentiality, a consent form and the questionnaire. Second and third reminders were sent out. The survey ran from 1 June 2021 until 1 October 2021. All data were stored in a password encrypted excel file.

### 2.2. Statistical Analysis

Data were analyzed using SPSS version 24 (IBM Corp. Released 2016. IBM SPSS Statistics for Windows, Version 24.0. Armonk, NY, USA: IBM Corp.). The mean and standard deviation (SD) were computed for the numeric values. The frequency and percentage were computed for the nominal variables, Pearson’s Chi-square test was used to determine any statistical differences in the participants’ response pattern and *p* < 0.01 level of significance was considered to test the level of significance.

## 3. Results

One hundred and fifty-three dentists completed questionnaires giving a 46.2% response rate with a 5.8% margin of error and a confidence level of 95%.

### 3.1. Characteristics of the Study Participants

**Figure 1:**
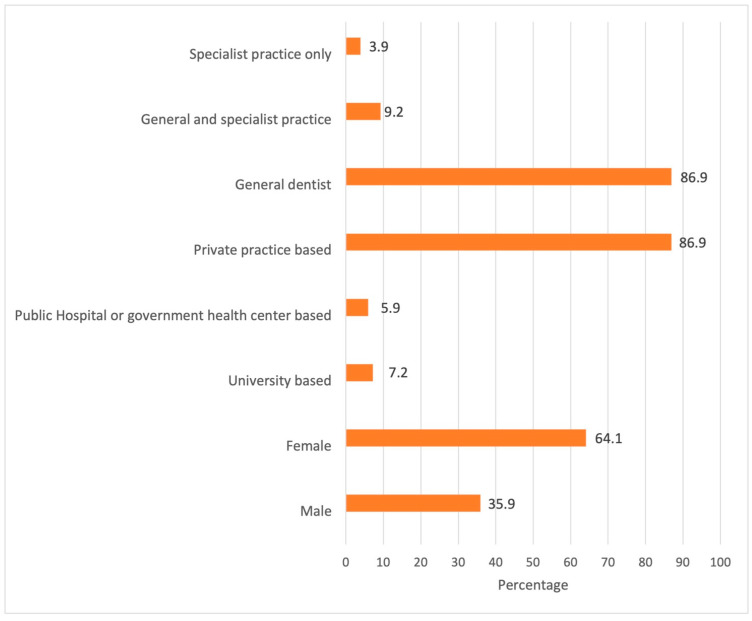
Characteristics of the selected sample in percentages.

Of the 153 respondents, 64.1% were female with an age range of 24 to 78 years: the mean age was 41.13 ± 11.68 years. A total of 86.9% of the respondents were general dentists, and the average practicing experience was 14.99 ± 10.97 years. A total of 86.9% of the respondents worked in private practice, 7.2% worked at the university and 5.9% at government health centres. These data are shown in Figure 1.

### 3.2. Dentists’ Knowledge Regarding COVID-19

The percentage of respondents who responded correctly to questions in the knowledge of COVID-19 section of the questionnaire were as follows: transmission of the virus—94.8%; persistence on surfaces—93.5%; transmission by asymptomatic patients—100%; hand hygiene—86.3%; use of PPE—98.7%; the need to minimize aerosol generating procedures (AGPs)—88.9%; use of N95 respirators when performing AGPs—93.5%; knowledge about the reuse of the N95 masks—27.5%; disinfection of surfaces with sodium hypochlorite—67.3%. All χ^2^ values presented below in Table 1 for the selected sample with regard to knowledge about COVID-19 were found to be statistically significant (*p* < 0.01).

### 3.3. Dentists’ Attitudes toward Treatment of Patients with COVID-19 and Other Concerns

A total of 34.9% of respondents were comfortable providing urgent or emergency care to patients, and 8.6% were comfortable providing elective dental treatment. A total of 80.3% were comfortable with the screening process for COVID-19. The fear of contracting COVID-19 from a patient was 64.5%, while 61.6% were afraid of contracting COVID-19 from work colleagues. A total of 24.4% of dental surgery assistants expressed a desire to stop working due to fear of contracting COVID-19. A total of 91.5% of respondents were concerned about the impact of COVID-19 on their livelihoods. All χ^2^ values presented below in Table 2 for the selected sample with regard to their attitude toward treatment of patients with COVID-19 and other concerns were found to be statistically significant (*p* < 0.01).

### 3.4. Dentists’ Practices

With regard to the use of N95 masks, 67.3% of respondents reported using them for all procedures, while 10.5% reported not using them at all. A total of 78.8% re-used N95 masks for multiple patient encounters without removing them, and 60.9 % changed them at the end of each day. A surgical mask was worn over the N95 mask by 55.6% of respondents. PPE was always utilized by 97.4% of dentists and 98.1% of dental surgery assistants. Hand hygiene was performed by 94.1% of respondents before donning gloves and after glove removal. Gloves were changed after each patient by 99.3% of respondents.

Instruments and hand pieces were sterilized after each patient by 98% of respondents; 100% reported disinfecting the patient’s chair, light and surfaces of the operatory after each patient, and 59.2% always disinfected all surfaces, chairs and doors of waiting areas every 2 h. A fallow period was always or sometimes utilized by 82.9% of respondents. Air purifiers were utilized by 37.2% of respondents, while 32.7% reported modifying the ventilation of the practice. All χ^2^ values presented below in Table 3 for the selected sample with regard to their practices were found to be statistically significant (*p* < 0.01).

### 3.5. Sources of Information Regarding COVID-19

**Figure 2:**
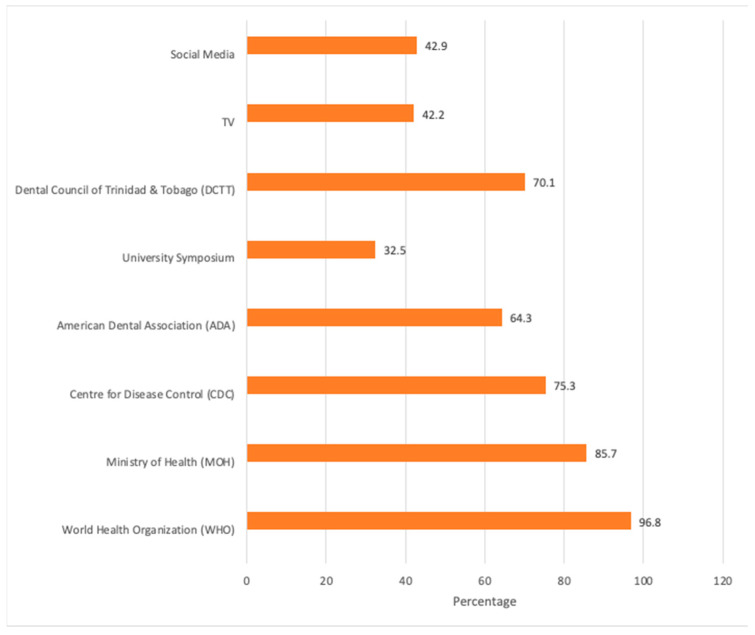
Sources of Information regarding COVID-19.

Respondents reported that their sources of information on COVID-19 came from WHO (96.8%); MOH (85.7%); CDC (75.3%); and DCTT (70.1%). These data are shown in Figure 2.

### 3.6. Dentists’ Attitudes/Acceptance towards COVID-19 Vaccines

A total of 90.8% of respondents stated that they will be vaccinated if a COVID-19 vaccine were made available to them “right now”. Those who responded no to this question wanted to ensure that the vaccine was safe.

A total of 90.8% of respondents agreed that COVID-19 vaccination would be an effective way to prevent and control COVID-19, while 38.6% were worried about possible side effects of the vaccine. Many respondents (81.6%) were willing to be trained to administer COVID-19 vaccines, and 86.1% were willing to have advocacy messaging/posters in their practice to encourage the uptake of COVID-19 vaccines in the population. All χ^2^ values presented below in Table 4 for the selected sample were found to be statistically significant (*p* < 0.01) with regard to their vaccine acceptance and attitude towards vaccines.

Further statistical analysis did not reveal any statistically significant differences with regard to dental knowledge, attitudes, practices and vaccine acceptance, and attitude towards vaccines for different subgroups such as sex or years of practicing experience.

## 4. Discussion

The present study provides an insight into the dentists’ knowledge, attitude and practices regarding the COVID-19 pandemic and vaccine acceptance in Trinidad and Tobago. At the time of the study, the university and regulatory body were providing online training and guidance for the management of patients and practice environment to mitigate the risk of spread of COVID-19. The majority of dentists in Trinidad and Tobago had good knowledge of COVID-19 (94.8%), use of PPE (98.7%), hand hygiene (86.3%) and use of N95 masks (93.5%). This level of knowledge is consistent with other studies in Lebanon (91.3%) [10], Pakistan (93.2%) [14], Taiwan (94.7%) [15], the Caribbean region [16] and other nations (92.7%) [12]. Other surveys showed adequate levels (83.1%) of awareness of COVID-19 infection [17], and 765 dentists in India 80.8% had adequate knowledge and preparedness [18]. In a multi-center study in Saudi Arabia, it was found that dentists’ knowledge scores were higher than undergraduate and postgraduate students [19]. The dentists in this survey were generally less knowledgeable (67.3%) about disinfection practices, which was similar to another survey where only 66.8% had adequate knowledge of disinfectants that should be used [11]. Some of the more recent studies investigating knowledge, attitudes and behavior conducted in 2021 (around the same time as our study) also showed good compliance with WHO and CDC guidelines [20] and good scores of knowledge and preventive practice [21], and are consistent with our findings. The high levels of knowledge and preventive practice may be due to the fact that our survey was conducted almost 15 months into the pandemic and most dentists were receiving their information from a variety of established sources at that time such as WHO (96.8%), CDC (75.3%) and the local Ministry of Health (85.7%).

The attitudes of dentists in this survey also revealed that only 34.9% were comfortable providing emergency care to positive or suspected cases of COVID-19, and high levels were uncomfortable (91.4%) providing elective care. This was in agreement with another multisite survey of over 1000 dentists from the North American, European, Eastern Mediterranean and Western Pacific regions that found low levels of comfort with preventive measures and provision of treatment [22]. Nearly 65% of dentists in this survey were afraid of becoming infected from a patient, which was similar to a survey in Pakistan with 75% being afraid [23]. There was also concern about their livelihood being affected, and similar concerns were found by dentists around the world [17,22].

In terms of dentists’ practices, the levels of usage of PPE including masks, gloves, goggles, face shields, gowns and N95 masks were high in this study (97.4%), which is consistent with findings in other studies [10,15] but at odds with a study from Iran (44%) [24], due to the shortage of supplies in that country at the time of that study. In terms of N95 mask usage, 78.8% always or sometimes used the N95 mask for multiple patient encounters in one day, and 60.9% always changed the N95 mask every day. These findings were similar to those found by [10,15,25], but only 38.5% of dentists wore N95 masks in another study in Pakistan [23]; in Saudi Arabia, only 40% considered N95 use necessary [26]; and only 12% used N95 masks in Turkey [20]. While 100% of dentists disinfected the chair and light after every patient in their surgery, with regards to practice and levels of disinfection of the waiting room every 2 h, only 59.2% of the dentists in this study always did it, which is similar to other reported findings [11] as well as another study in Lebanon (58.7%) [10]. The guidance offered by the DCTT might explain the good levels of preventive practice reported.

The vast majority of dentists agreed that COVID-19 vaccination would be an effective way to prevent and control COVID-19, and 90.8% had agreed to be vaccinated straight away if a vaccine was available; however, 38.6% were worried about the side effects. Over 80% were willing to be trained to administer vaccines. This study showed high levels of vaccine acceptance among this sample of dentists in Trinidad and Tobago. Vaccine hesitancy is a public health issue worldwide [27,28], and has also been shown to be an issue among healthcare workers (HCWs) [13]. In a study in Israel, dentists showed less hesitancy to be vaccinated against COVID-19 than dental hygienists [29]. Another study in the USA found 45% of dental students were hesitant about receiving the COVID-19 vaccine, while 23% of medical students were hesitant [30]. Dentists from private, government and academic clinics in Indonesia agreed that vaccination makes an individual healthier and safer from COVID-19 infection [31]. Our study shows high levels of vaccine acceptance, which was similar to other studies amongst dentists in Lebanon (86%) [32], Israel (85%) [33] and Greece (82.5%) [34]. This could be explained by the fact that the survey occurred a few months after vaccines had become available and been administered to persons in the UK, USA and Europe, which was observed in the media—perhaps providing some reassurance in terms of safety. However, some other studies on HCWs in the Congo [35] and USA [36] showed low levels of vaccine acceptance, 27.7% and 36%, respectively, which could be due to the surveys being conducted early on in the pandemic in April and October 2020, respectively, when vaccine information was new and surrounded with uncertainty. A multi-country survey of dental professionals conducted in February–April 2021 found 72.5% of participants reporting their intention to accept the vaccine; however, there was significant difference in agreement across countries [37].

The government of Trinidad and Tobago was able to provide a free COVID-19 vaccine, AstraZeneca from the COVAX facility, starting in June 2021. The Sinopharm vaccine then became available in the country from July 2021. The high response of dentists’ willingness to be trained to administer vaccines actually materialized, as the dentists were able to take part and administer vaccines in the first phase of the government rollout program of vaccinations, as at the time they were limited to only emergency procedures in their practice. Under the period of public health emergency proclaimed on 12 June 2021, dentists and dental interns were able to administer a COVID-19 vaccine under the supervision of a medical practitioner. The dentists provided great voluntary service to the country providing much needed manpower in the initial rollout of the vaccination program and had high rates of willingness for advocacy of COVID-19 vaccines. Vaccine acceptance is influenced by factors such as perceived risk and efficacy [38], and while Trinidad and Tobago is very diverse culturally and ethnically, it has been shown that the public requires clear communication and transparency about vaccination programs in order to enhance trust and acceptance of vaccines [39]. A vaccine hesitancy survey conducted in October and November 2021 stated that 65% of the general population said that they had been vaccinated against COVID-19 [40]. Despite these survey findings, only 51.3% of the population (718, 124 persons) are fully vaccinated to date according to actual government figures (MOH, GOVRTT 20 December 2022) [5]. While vaccine acceptance is high amongst dentists in Trinidad and Tobago, vaccine hesitancy within the general population of Trinidad and Tobago appears to still remain a concern, and further research is required in this area.

This study has limitations, as the response rate was less than 50% and non-response bias can affect the generalizability of the study. The study was conducted via social media and email, and some dentists may not have had access and thus been excluded from the survey. Additionally, there may be self-reported data that may not be accurate, and an overall comparison to other studies with different questionnaire items may also be more general than exact. The authors acknowledge that the study was conducted after government and DCTT recommendations were given, which might influence respondents high level of knowledge. If this is the case, the results might be more reflective of the level of responsiveness and acceptance of the recommendations given.

## 5. Conclusions

Dentists in Trinidad and Tobago have good levels of knowledge, attitude and practices regarding COVID=19, and appear to be consistent with the recommended guidelines for COVID-19 infection control in dental practices. There were some shortcomings in the area of waiting room disinfection and also the reuse of N95 masks. Dentists have high levels of vaccine acceptance, and findings from this study can guide improvements in continuing to maintain high levels of infection control to prevent the spread of COVID-19 from patients. Finally, dentists can play a role in advocating for the uptake of the COVID-19 vaccine.

## Figures and Tables

**Table 1:** Dentists’ knowledge about COVID-19.

Sr. No	Items	Correct	Incorrect	Not Sure	Chi Square
1.	COVID-19 is transmitted through direct contact with respiratory tract secretions	145 (94.8)	8 (5.2)	0 (00)	122.673
2.	COVID-19 can persist on surfaces up to a few hours and up to several days	143 (93.5)	8 (5.2)	2 (1.3)	249.294
3.	The disease can be transmitted from asymptomatic patients	153 (100)	0 (00)	0 (00)	NA
4.	The use of personal protective equipment (PPE) including masks, gloves, gowns, goggles or face shields is recommended to protect skin and mucosa from (potentially) infected blood or secretions	151 (98.7)	1 (0.7)	1 (0.7)	294.118
5.	Hand hygiene has been considered the most critical measure for reducing the risk of transmitting of coronavirus to patients	132 (86.3)	17 (11.1)	4 (2.6)	194.627
6.	All surfaces contaminated by the patient with COVID-19 should be disinfected with sodium hypochlorite (5% bleaching solution)	103 (67.3)	27 (17.6)	23 (15)	79.686
7.	Dentists should avoid or minimize aerosol- generating procedures (AGPs)	136 (88.9)	9 (5.9)	8 (5.2)	212.510
8.	PPE Donning sequence (1) gown (2) mask (3) gloves	129 (84.3)	18 (11.8)	6 (3.9)	180.353
9.	PPE Doffing (removal) sequence (1) gloves (2) gown (3) mask	116 (75.8)	27 (17.6)	10 (6.5)	127.098
10.	Is the use of N95 respirators (masks) or European Standard Filtering Face Piece 2 (EU FFP2) recommended for when performing aerosol=generating procedures (AGPs)?	143 (93.5)	4 (2.6)	6 (3.9)	248.980
11.	Is it recommended to reuse the N95 respirators (masks)?	42 (27.5)	85 (55.6)	26 (17)	36.510

**Table 2:** Dentists’ Attitude toward Treatment of Patients with COVID-19 and other concerns.

Sr. No	Items	Yes	No	N	Chi Square
1.	I am comfortable providing urgent or emergency care in my clinic to a known COVID-19-positive patient or suspected case.	53 (34.9)	99 (65.1)	152	13.921
2.	I am comfortable providing elective treatment in my clinic to a known COVID-19-positive patient or suspected case.	13 (8.6)	139 (91.4)	152	104.447
3.	I am comfortable with the screening process at my clinic to detect potential COVID-19 cases.	122 (80.3)	30 (19.7)	152	55.684
4.	I am afraid of becoming infected with COVID-19 from a patient.	98 (64.5)	54 (35.5)	152	12.737
5.	I am afraid of becoming infected with COVID-19 from a work colleague.	93 (61.6)	58 (38.4)	151	8.113
6.	My dental surgery assistant (DSA) expressed his/her desire to stop work or actually stopped work due to fear of infection with coronavirus.	31 (20.4)	121 (79.6)	152	53.289
7.	I am afraid of the impact of the COVID-19 crisis on dentists’ livelihood.	140 (91.5)	13 (8.5)	153	105.418
8.	The COVID-19 pandemic has negatively impacted my practice’s profitability since the relaxation of govt restrictions.	126 (83.4)	25 (16.6)	151	67.556

**Table 3:** Dentists’ practices.

Sr. No	Items	Always	Sometimes	Not at All	N	Chi Square
1.	Do you use N95 respirators (masks) or equivalent for aerosol-generating procedures (AGPs) in your clinic?	103 (67.3)	34 (22.2)	16 (10.5)	153	82.706
2.	Do you use the same N95 masks for multiple patient encounters without removing the mask between different patient encounters?	63 (41.7)	56 (37.1)	32 (21.2)	151	10.503
3.	Do you change your N95 after each day?	92 (60.9)	38 (25.2)	21 (13.9)	151	54.609
4.	Do you re-use the N95 mask on another day?	32 (21.2)	56 (37.1)	63 (41.7)	151	10.503
5.	Do you sterilize your used N95 masks?	34 (22.7)	35 (23.3)	81 (54)	150	28.840
6.	Do you double mask, i.e., use a regular surgical mask over your N95 mask?	84 (55.6)	32 (21.2)	35 (23.2)	151	33.868
7.	Do you change gloves after every patient?	152 (99.3)	1 (0.7)	0 (00)	153	149.026
8.	Do you perform hand hygiene before putting on gloves and immediately after removing gloves?	144 (94.1)	8 (5.2)	1 (0.7)	153	254.863
9.	Do you disinfect the patient’s chair and light and surfaces of the operatory after each patient?	152 (100)	0 (00)	0 (00)	152	NA
10.	Do you (dentist) wear the personal protective equipment (PPE) such as gloves, masks, goggles or face shields, head cover and foot cover for aerosol-generating procedures (AGPs)?	140 (91.5)	9 (5.9)	4 (2.6)	153	233.216
11.	Does your dental assistant (DSA) wear the personal protective equipment (PPE) such as gloves, masks, goggles or face shields, head cover and foot cover for aerosol-generating procedures (AGPs)?	141 (92.2)	9 (5.9)	3 (2)	153	238.588
12.	Do you disinfect all surfaces, chairs and doors of the waiting area every 2 h?	90 (59.2)	45 (29.6)	17 (11.2)	152	53.539
13.	Do you give separate appointments and avoid patients congregating in the waiting room?	138 (91.4)	11 (7.3)	2 (1.3)	151	229.841
14.	Do you sterilize instruments and hand pieces after each patient use?	150 (98)	3 (2)	0 (00)	153	141.235
15.	Do you utilize a fallow period?	68 (46.6)	53 (36.3)	25 (17.1)	146	19.575
16.	Have you installed any air purifiers in your practice?	55 (37.2)	16 (10.8)	77 (52)	148	38.689
17.	Have you modified your practice’s ventilation/air flow?	48 (32.7)	30 (20.4)	69 (46.9)	147	15.551

**Table 4:** Dentists’ vaccine acceptance and attitudes towards vaccines.

				Yes	No	Chi Square
If a free WHO-approved vaccine to prevent COVID-19 was available right now, would you agree to be vaccinated?				139 (90.8)	14 (9.2)	102.124
If your answer is no to above question, then what is the main reason that you would not agree to receive a coronavirus/COVID-19 vaccine?						
Concerns about rushed timeline					2 (1.3)	
Do not trust vaccines					1 (0.7)	
Want to wait to confirm how effective it is					3 (2.0)	
Want to wait to confirm it is safe					8 (5.2)	
**Sr. No**	**Items**	** Agree **	** Disagree **	** Undecided **	**N**	**Chi Square**
1.	COVID-19 vaccination is an effective way to prevent and control COVID-19.	139 (90.8)	1 (0.7)	13 (8.5)	153	229.176
2.	I am worried about possible side effects of the vaccine.	59 (38.6)	67 (43.8)	27 (17.6)	153	17.569
3.	I am healthy so I do not need to take the vaccine.	3 (2)	145 (95.4)	4 (2.6)	152	263.461
4.	I am more likely to take a vaccine if I need it to travel to other countries.	69 (45.7)	73 (48.3)	9 (6)	151	51.073
5.	I do not need a vaccine because I have had a positive COVID-19 test.	6 (4)	139 (92.1)	6 (4)	151	234.291
6.	I would be willing to be trained to administer COVID-19 vaccines	124 (81.6)	11 (7.2)	17 (11.2)	152	159.566
7.	I would be willing to have advocacy messaging /posters in my practice to encourage the uptake of COVID-19 vaccines in the population.	130 (86.1)	4 (2.6)	17 (11.3)	151	190.821

## Data Availability

Not applicable.
